# Efficient Low Shear Flow-based Trapping of Biological Entities

**DOI:** 10.1038/s41598-019-41938-z

**Published:** 2019-04-02

**Authors:** Ahmad Sohrabi Kashani, Muthukumaran Packirisamy

**Affiliations:** Optical Bio Microsystem Lab, Mechanical, Industrial, and Aerospace Engineering Department, Concordia University, Montreal, Quebec, H3G 1M8 Canada

## Abstract

Capturing cells or biological entities is an important and challenging step toward *in-vitro* studies of cells under a precisely controlled microscale environment. In this work, we have developed a compact and efficient microdevice for on-chip trapping of micro-sized particles. This hydrodynamics-based trapping system allows the isolation of polystyrene micro-particles with a shorter time while inducing a less hydrodynamic deformation and stress on the particles or cells both after and before trapping. A numerical simulation was carried out to design a hydrodynamic trapping mechanism and optimize the geometric and fluidic parameters affecting the trapping efficiency of the microfluidic network. By using the finite element analysis, the velocity field, pressure field, and hydrodynamic force on the micro particles were studied. Finally, a PDMS microfluidic device was fabricated to test the device’s ability to trap polystyrene microspheres. Computational fluid analysis and experimental testing showed a high trapping efficiency that is more than 90%. This microdevice can be used for single cell studies including their biological, physical and chemical characterization.

## Introduction

In recent years, the use of microfluidics, and microfabrication has been adopted in the various areas of engineering and science. Microfluidics is an important technology, suitable for numerous biomedical application fields such as drug delivery, cellular analysis, diagnosis, cancer detection, and biotechnology^1,2^. One of the most exciting advancements in the application of microfluidics has happened in the biological and biomedical areas. Reduced sample volume, short time analysis, control of spatio-temporal chemical composition, precise and predictable fluid flow regime (laminar flow), portability and integration with sensors actuators, controllers and automation systems have made microfluidic devices an attractive miniaturized platform for biological and biomedical applications^3,4^.

Capturing and trapping of cells is the first critical step in single cell studies and can be defined as development of technologies for the immobilization of cells at a precise location for further analysis. At the same time, a fluid flow needs to be applied to provide the cells with nutrients and other media influencing the behavior of the cells. Isolation of single cells from the surrounding environment and their analysis have important implications for diagnosis and therapy. For example, capturing circulating cancer cells in peripheral blood is beneficial for detecting cancer at early stages and estimating the risk of metastatic relapse^5–7^. Before the advancement of microfluidics, conventional strategies such as sample tubes and pipettes were used to manipulate the biological particles at the microscale. The lack of sufficient precision, high-throughput, online monitoring, time and reagent consuming have limited their application in single cell analysis. Many microfluidic-based technologies have been developed to control the spatio-temporal of the environment of cells. Based on the types of forces used in the manipulation of the cells on the chip, they are classified into different categories: optical^8^, electrical^9^, magnetic^10^, acoustic^11^ and hydrodynamic manipulations^12^. Hydrodynamic trapping of cells offers a low-cost, simple, efficient and high-throughput arraying of the cell without controlling or using other equipment such as lasers, electrodes, magnets, and ultrasound transducers. In this technique, the sample and the medium are introduced by flow channels providing conditions for the observation of the responses of the cells to the environmental changes^13,14^. Depending on whether the target particles are in contact with a support surface, the hydrodynamic trapping systems are classified into two main methods: contact-based methods^15^ in which the fluid flow is implemented to physically confine the cells against microfabricated obstacles or walls, and contact-less methods^16^ which are based on micro eddies, micro-vortices and stagnation flow. Due to some limitations of contact-less trapping technique such as low efficiency and low-throughput, most of the efforts have been directed toward the implementation of the contact-based methods to capture and manipulate individual cells. Two concepts have been used for the contact-based trapping technique: (1) Path with the least flow resistance^17^ where single cells are directed into the trap sites possessing much smaller hydrodynamic resistance compared to the bypass or the main path, and after filling the trap site, the subsequent cell is redirected to another path having the least flow resistance, (2) Di Carlo^18^ method, in which micro-sieve arrays are designed to trap single cells from a suspension of flow. This method suffers from a relatively low-efficient single-cell trapping compared to the first concept and most of the samples are lost during the trapping process. In order to optimize the efficiency of hydrodynamic single-cell capturing systems, the geometric parameters, as well as the fluid flow factors, should be taken into account.

Here, we developed a lab-on-a-chip system using the contact-based hydrodynamic trapping method to immobilize single cells by positioning a series of micro-sieves on the main channel sidewalls. In this system, the combination of the two above-mentioned concepts is implemented to design an efficient trapping microfluidic device. In our design, similarly to Di Carlo design, micro-sieves are used, but on the sidewalls, to capture individual cells or particles while at the same time the concept of “path with the least resistance” is utilized to tune the geometric parameters of the main and the side channels in order to enhance capturing efficiency of the device. The network includes two side channels and a converging main channel designed to direct the streamlines toward the trapping sites. In contrast to Tan and Takeuchi^17^ and other works, a channel with inclined walls is designed to increase the hydrodynamic resistance of the main channel, enabling to navigate single cells into the trapping sites. With the proposed design, the trapping device can be more compact as the main channel does not need to be extended to generate a higher flow resistance than trap sites. Compared with the previous studies using a similar concept, this design offers a fast loading time while less mechanical deformation and stress are introduced onto the target cells both before and after trapping. In this work, first, we used a computational fluid dynamic simulation analysis by COMSOL Multiphysics to optimize the geometric parameters of the channels and trap sites, and predict the fluid flow pattern inside the channels, considering the dimensions of the target biological particles. Finally, polystyrene beads were used in order to demonstrate the feasibility and the working mechanism of the fabricated device to simulate the capture of single cells.

## Conceptual Design of the Device

**Figure 1:**
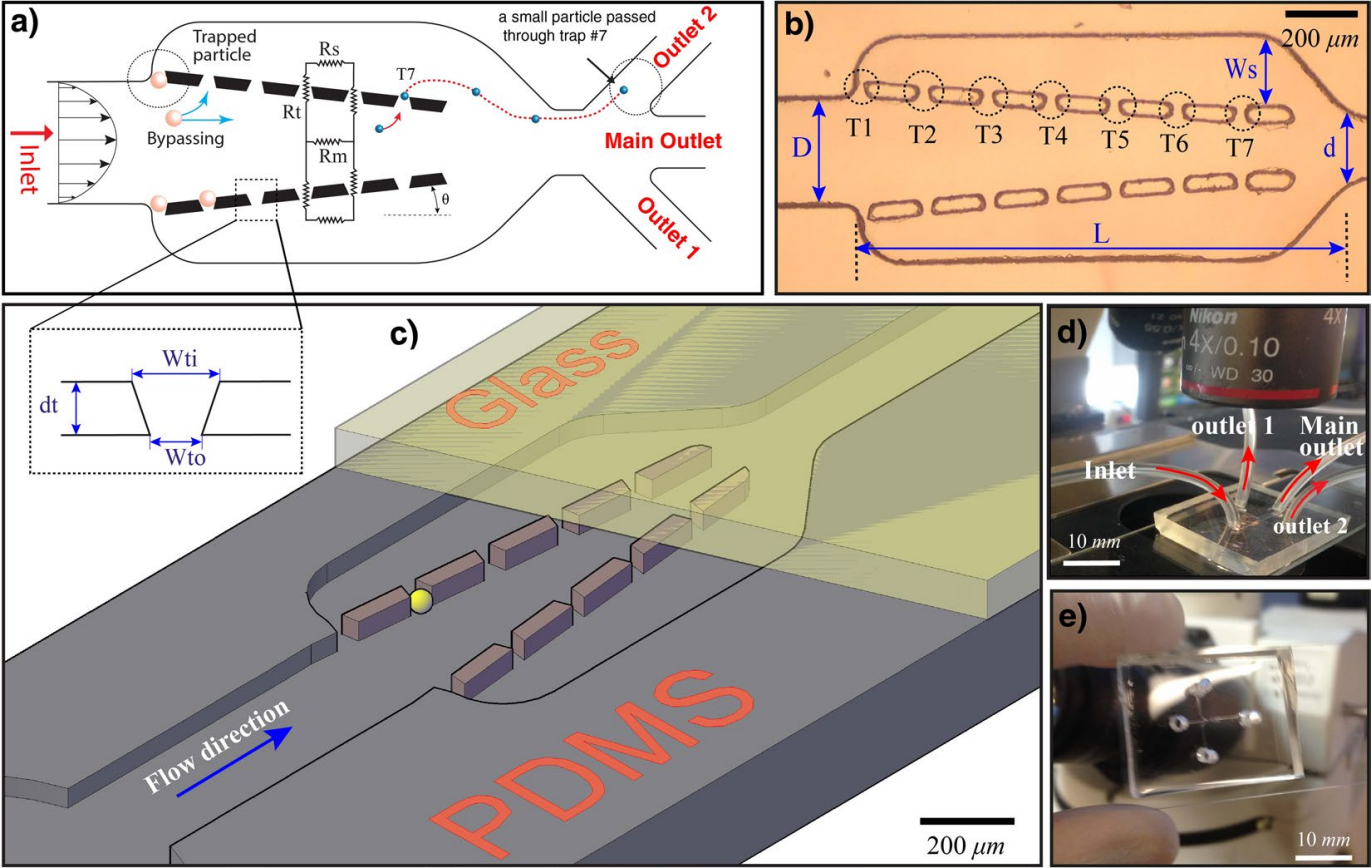
(**a**) Schematic illustrating the working concept of the system and the fluidic resistances of the main channel (R_m_), side channels (R_s_) and trap sites (R_t_) and the dimensions of the trap: inlet of the trap width (W_ti_), outlet of the trap width (W_to_), depth of each site (d_t_), (**b**) Micrograph of the microfluidic showing the geometric parameters of the main channel and side channels (D: inlet of the main channel, d: outlet of the main channel, L: length of the main channel, and W_s_: maximum width of the side channel), (**c**) 3D illustration of the trapping device, (**d**) The PDMS microfluidic device and the inlet and outlet connections, (**e**) A photograph of the fabricated chip.

The illustration of the trapping system is shown in Fig. 1. The microfluidic arrangement is composed of a main channel, trap sites and two side channels connected to the main channel at the outlet. The trapping sites are shaped in trapezoid grooves, having the outlet opening smaller than target particles, to capture the particles. The concept of this design is to keep the flow resistance of the empty sites low compared to the main channel in order to direct the particles into the trap sites through optimizing the geometry of the traps and the main channel. The design contains many hydrodynamic trap sites placed between the side and the main channels. The main objective of the design is to reduce the number of unoccupied sites and maximize the trapping efficiency of each trap and let the extra cells or particles to be drained by the outlet of the main channel in order to avoid blocking the main channel. Also, the main and side channels need to be designed such that each trap achieves not more than a single particle/cell while they experience a low hydrodynamic stress. Each entering particle from the inlet of the main channel can be either transported by the medium to one of the trap sites or can be drained by the outlet of the main channel. Assuming laminar flow inside the microfluidic channel (without gravitational and inertial forces) and when there are no interactions between particles and microfluidic walls, each particle follows the streamlines, and the efficiency of trapping can be characterized by the flow rate through trap sites^19^. When the particles’ Reynolds number is very low (<1), particles follow the flow streamlines. Microparticles/cells are in micro scale and the inertial effects which cause particles to travel across streamlines are very small compared to the viscous force, and they tend to follow streamlines^20^. The flow rate through each path can be determined by the corresponding hydrodynamic resistance. In fact, more flow can be passed through the path with a lower hydrodynamic resistance. The hydrodynamic resistance of the empty trap sites, main channel, and the flow parameters (input and output conditions) of the network need to be controlled to manage the trapping efficiency of the device and the fluid pressure difference between the main and side channels. In order to increase the efficiency of trapping, either the hydrodynamic resistance of the main channel has to be increased or the hydrodynamic resistance of the trapping path has to be reduced. The dimension of trap sites depends on the size of target cells, and the dimension of trap sites need to be small enough to not allow the target cells to pass through them. Due to the size constraints of the traps, only limited changes on the dimensions of the trap sites can be performed. As a result, to optimize the trapping efficiency of the system and to maximize the flow rate in the traps, one has to manipulate the geometrical parameters of the other paths such as the main and the side channels. The hydrodynamic resistance (R) of a channel with an arbitrary cross-section can be obtained from the following relationship^21^:

1 $$R=\frac{{\rm{\Delta }}P}{Q}=16{\pi }^{2}\mu \,{\int }_{0}^{L}\frac{{I}_{p}(x)}{A{(x)}^{2}}dx+\rho Q(\frac{1}{{A}_{2}^{2}}-\frac{1}{{A}_{1}^{2}})$$

**Figure 2:**
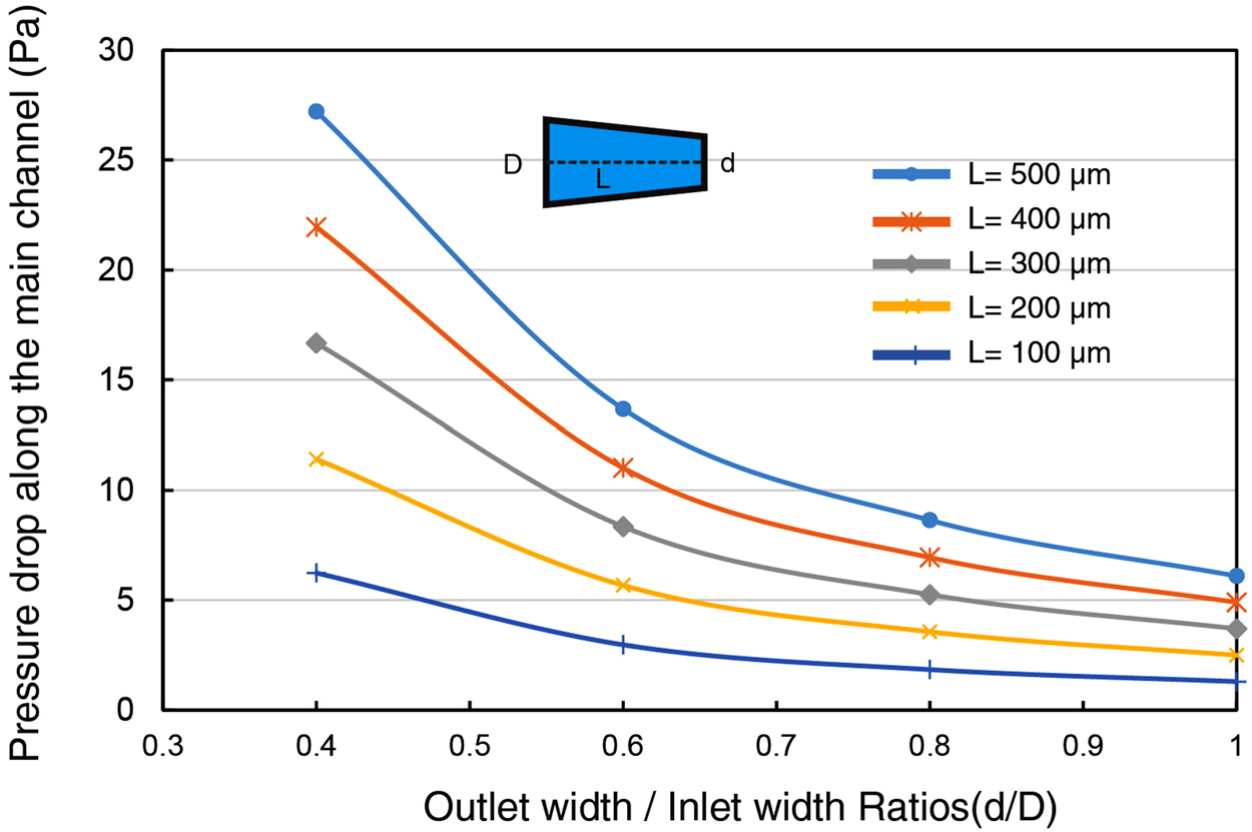
Pressure differences along a channel with different lengths and d/D ratios (The inlet velocity: 2.8 mm/s, D: inlet width of the main channel, d: outlet width of the main channel).

Where A_1_ and A_2_ are the microchannel cross-section area of the inlet and outlet of the channel respectively, L is the length of microchannel, Q is the flow rate inside the channel, Δ*P* is the pressure difference along the microchannel, µ is the dynamic viscosity of fluid, and *I*_*p*_ is the specific polar momentum of inertia. From this equation, it is obvious that the fluidic resistance of a certain channel is not only a function of its geometry but also is proportional to the flow rate inside the channel. For a constant cross-sectional area channel, the area of inlet and outlet are same, so that the last term of eq. 1 can be canceled and the hydrodynamic resistance of the straight channel becomes purely the function of geometry such as length, height, and width^22^. Increasing the fluidic resistance along the main channel is one of the simple ways to increase the trapping efficiency of the system^23^. In order to increase the hydrodynamic resistance of the system, the straight main channel can be replaced by a rectangular micro-channel cross-section with a linear wall profile, allowing to enhance the pressure gradient between the main and side channels. Using a converging micro-channel, the last term in eq. 1 increases due to the reduced area at the outlet, producing a greater hydrodynamic resistance at the main path. Without changing the angle of side walls and only by increasing the length of the main channel, the hydrodynamic resistance of the main channel can also be increased to provide a positive pressure gradient toward trap sites(According to the Darcy-Weisbach and for the Hagen- Poiseulle flow, the flow resistance is directly proportional to the length of the channel and the longer is the main channel, the larger will be the flow resistance). However, with the aid of a converging channel not only the microsystem can be more compact, but the particle loading time can be faster as well. Figure 2 shows pressure difference along a converging channel for different lengths of the channel and the different ratio of outlet to inlet width of the main channel (*d*/*D*). The length of a straight channel with the equivalent flow resistance is presented in Table 1. For instance, for designing a straight channel with a flow resistance as same as a converging channel with *d*/*D* = 0.4, and *L* = 100 *μm*, the length has to be extended 5.2 times compared to the length of the converging channel (*L*_*eq*_ = 5.2 × 100 = 520 *μm*).

**Table 1:** The ratio of an equivalent straight channel length to the converging channel length (*L*_*eq*_/*L*) for different d/D and L to produce the similar flow resistance.

Channel length(L)	d/D = 1	d/D = 0.8	d/D = 0.6	d/D = 0.4
100 µm	1	1.53	2.48	5.2
200 µm	1	1.49	2.37	4.76
300 µm	1	1.46	2.32	4.63
400 µm	1	1.45	2.30	4.58
500 µm	1	1.44	2.28	4.54

## Materials and Methods

### Computational analysis

A 3D finite element analysis (FEA) was performed to estimate the velocity field and pressure gradient within the microfluidic device. Incompressible Naiver-Stokes and continuity equations were employed to characterize fluid flow parameters inside the micro channels^24^.

2 $$\rho \frac{\partial u}{\partial t}-{\rm{\Delta }}(-pI+\mu (\nabla u+{(\nabla u)}^{T})+\rho (u.\nabla ).u=F$$

3 $$\nabla .(u)=0$$

Where ρ is the density of the fluid, p is the fluid pressure, u is the velocity field within the channel, I is unit diagonal matrix, µ is dynamic viscosity of fluid, and F is the volume force on the fluid that can be ignored for a pressure driven fluid flow in the absence of the other volume force. COMSOL Multiphysics® 5.2 was employed to solve the governing equations and determine the optimal geometrical parameters of the initial design in order to have an efficient hydrodynamic trapping system. In this model, a constant velocity was considered as the inlet boundary condition, and a uniform zero gauge pressure was set at the outlet of the main channel. The no-slip condition was applied to all the other surfaces. The suspended particles were considered as solid spheres, and the interaction between them was assumed to be negligible by considering the relatively low concentration of particles inside the channel. The trajectories of the particles entering the main channel were characterized using a numerical simulation to assess the trapping efficiency of the device. For a rigid particle moving through a fluid, the equation of motion is defined by the following equation^25^.

4 $$m\frac{d{u}_{r}}{dt}={F}_{e}-{F}_{b}-{F}_{D}$$

Where m is the mass of particle, *u*_*r*_ = *u* − *u*_*p*_ is the relative particle velocity to the fluid velocity, *u*_*p*_ is the particle velocity, *F*_*e*_ is external force and *F*_*b*_ is the buoyancy force. The gravitational force (including the buoyancy) is considered to be negligible and no external force is applied on particles. The drag force acting on the particles can be obtained from the following equation^26^

5 $${F}_{D}=\frac{{C}_{D}{u}_{r}^{2}\rho {A}_{p}}{2}$$

Where *C*_*D*_ is the drag coefficient, *A*_*p*_ is the projected area of the particle in the plane perpendicular to the flow direction. The drag coefficient is a function of particle Reynolds number defined as:

6 $$R{e}_{p}=\frac{\rho |u-{u}_{p}|{d}_{p}}{\mu }$$

where *d*_*p*_ is the diameter of particles. For creeping motion (Stokes flow) when *Re*_*p*_ < 1, the inertia force is negligible and the viscous force is the predominant force^27,28^, the particles follow the fluid streamlines^19^. Considering the maximum velocity of particles in trap sites, the particle’s Reynold number for the inlet velocity of 2.8 mm/s is less than 0.1, and remains less than unity, for the inlet velocity of 28 mm/s, confirming that for this range, particles indeed follow the streamlines with good accuracy.

### PDMS chip Fabrication

Conventional soft lithography was used to fabricate the PDMS microfluidic chips. AutoCAD was used to draw the design layouts which were printed onto the masks (Fineline Imaging, Colorado Spring, USA). Photolithography was used to fabricate the master molds on silicon wafers. The negative photoresist (SU-8 2035) was spin coated at 1900 rpm for 30 s on a 4″ silicon wafer to obtain a layer with 70 μm thickness. Before exposing it to the UV, the photoresist was baked at 65 °C for 3 min and at 95 °C for 6 min. The transparent mask was then used to pattern the design using a mask aligner and UV light for 20 s and post-baked at 65 °C for 2 min. and at 95 °C for 7 min. In the final step, the SU-8 layer was developed for 5 min using SU-developer to obtain the mold and finally it was dried with an N2 gun. Before casting the PDMS, the surface of the mold was salinized in vapor phase for removing easily the cured PDMS. A mixture of pre-polymer and curing agent with a weight ratio of 10:1 was degasified in a vacuum desiccator to remove the bubbles. The PDMS was then poured onto the mold and placed on at 80 °C oven for 2 hours. Then, the PDMS layer was peeled off the mold, and the inlet/outlet were punched by a 1 mm punch. Finally, with the aid of oxygen plasma bonding, the PDMS chip was bonded to a glass slide to seal the channels.

### Bead Preparation

Polystyrene particles (Microspheres- Nanospheres, Cold Spring, NY) with a diameter of 50 μm were dispersed in 0.05% Tween 20 in Phosphate buffered saline (PBS) in order to have a solution with a concentration of 50 particles/μl. The suspension was sonicated to ascertain a good dispersion.

### Experimental Setup

The microfluidic chip consists of one inlet for injecting the medium and the particles suspension, and three outlets, two from the side channels and one from the main channel. A 2 ml syringe with 23 G needle was connected to the inlet of the fabricated device through a polytetrafluoroethylene (PTFE) tube with size of 0.59 *mm ID* × 0.25 *mm* thickness. Before using the microfluidic chip for operation, the PDMS surfaces were incubated for 30 min with tween 20 (10% in PBS) in order to prevent the polystyrene particles from binding to the channel surfaces. Tween 20 can effectively disrupt the hydrophobic interaction between the particles and the PDMS surfaces^29^. The inlet tube then was connected to the syringe pump (KD Scientific Legate 110) for loading the suspended microparticles into the device at a specific flow. The trapping process was next observed under a microscope (Nikon Eclipse 80i). A digital camera (Infinity) coupled to the inverted microscope was used to capture photographs and videos.

## Results and Discussion

### Simulation Results

The fluid flow in the design shown in Fig. 1 was simulated to understand the flow characteristics and to optimize the parameters affecting the trapping performance of the individual trap sites. The amount of flow passing each trap determines the trapping efficiency for various geometric configurations of the main and the side channels. By increasing the hydrodynamic resistance of the main channel, particles and cells have more chances to be trapped by side traps. The higher pressure gradient across side traps causes more flow through each trap resulting in a higher probability of trapping. The dimensions of each trap site were chosen in accordance with the diameter of the cells or particles. The inlet and outlet openings of each trap were set close to the particle diameter (1.2 and 0.8 times of the particle diameter, respectively), and the height of the microfluidic channels was set at 1.4 times the diameter, allowing particles to move freely within the microfluidic network. The width of the main inlet channel was selected large enough to let particles enter the trapping area without accumulation. The different features of the trap sites, the main channel and side channels for the three various designs are shown in Table 2.

**Table 2:** The geometric parameters of microfluidic network and trap sites (unit in μm).

Parameters	*d*/*D*	*d*	*W*_*s*_	*W*_*ti*_	*W*_*to*_	*d*_*t*_	*θ*
Design 1	1	350	150	60	40	40	0
Design 2	0.7	250	220	60	40	40	2.2°
Design 3	0.48	168	240	60	40	40	4.4°

**Figure 3:**
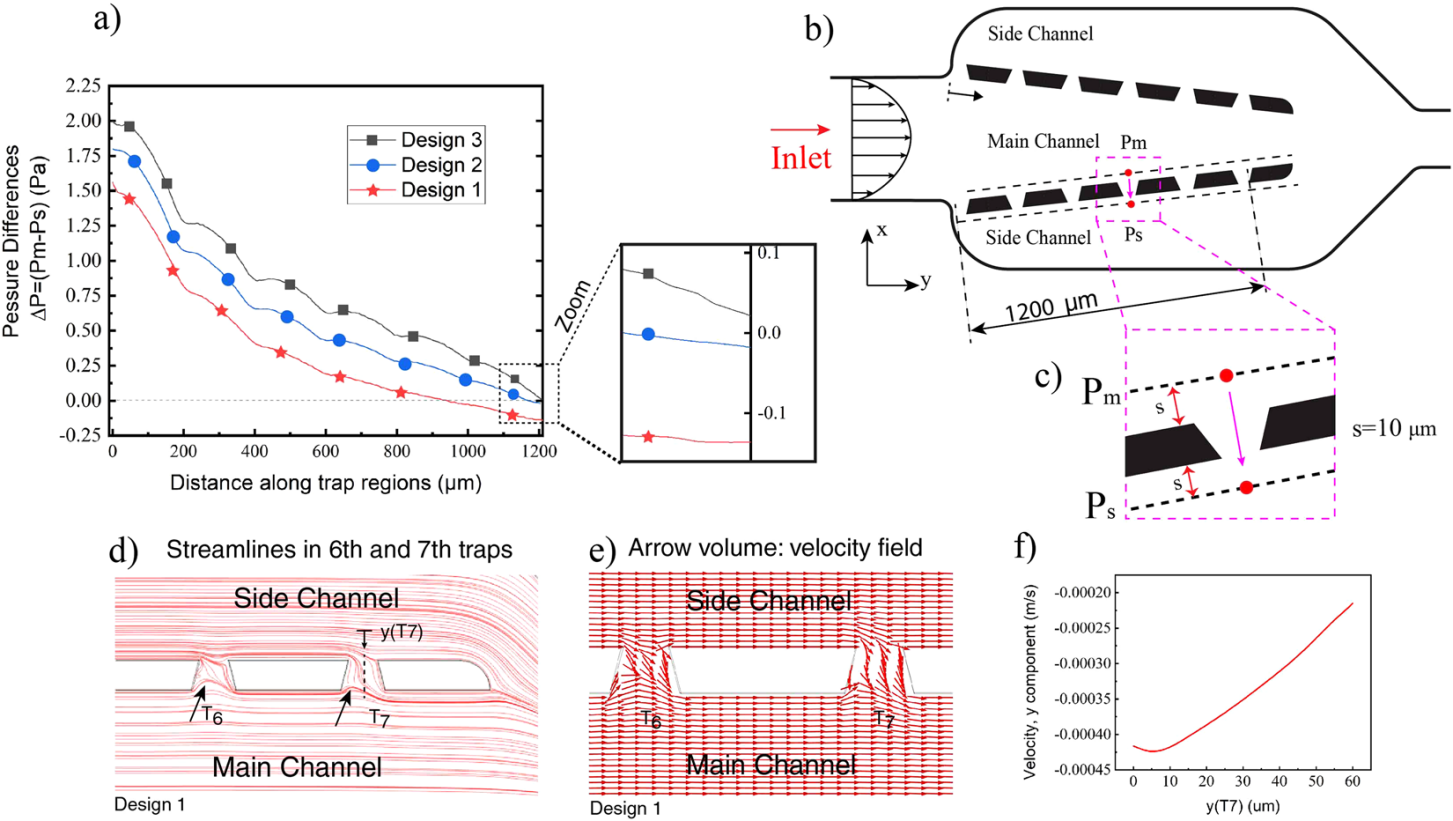
(**a**) Pressure difference (Δ*P* = *Pm* − *Ps*) between the main channel (*P*_*m*_) and side channels (*P*_*s*_) for three different designs, (**b**,**c**) schematic of main and side channels and traps and the coordinate for measuring Pm and Ps., (**d**) The flow streamlines within trap 6 and 7 in Design 1, showing the flow direction from the side channels to the main channel. Two arrows in 6^th^ and 7^th^ show that the flow is trying to enter the trap, but the gradient pressure is not strong enough to change the direction, (**e**) velocity field (normalized arrows) within 6^th^ and 7^th^ trap sites showing the flow direction in the traps, (**f**) velocity profile (v: in y direction) along a line passing through the last trap.

**Figure 4:**
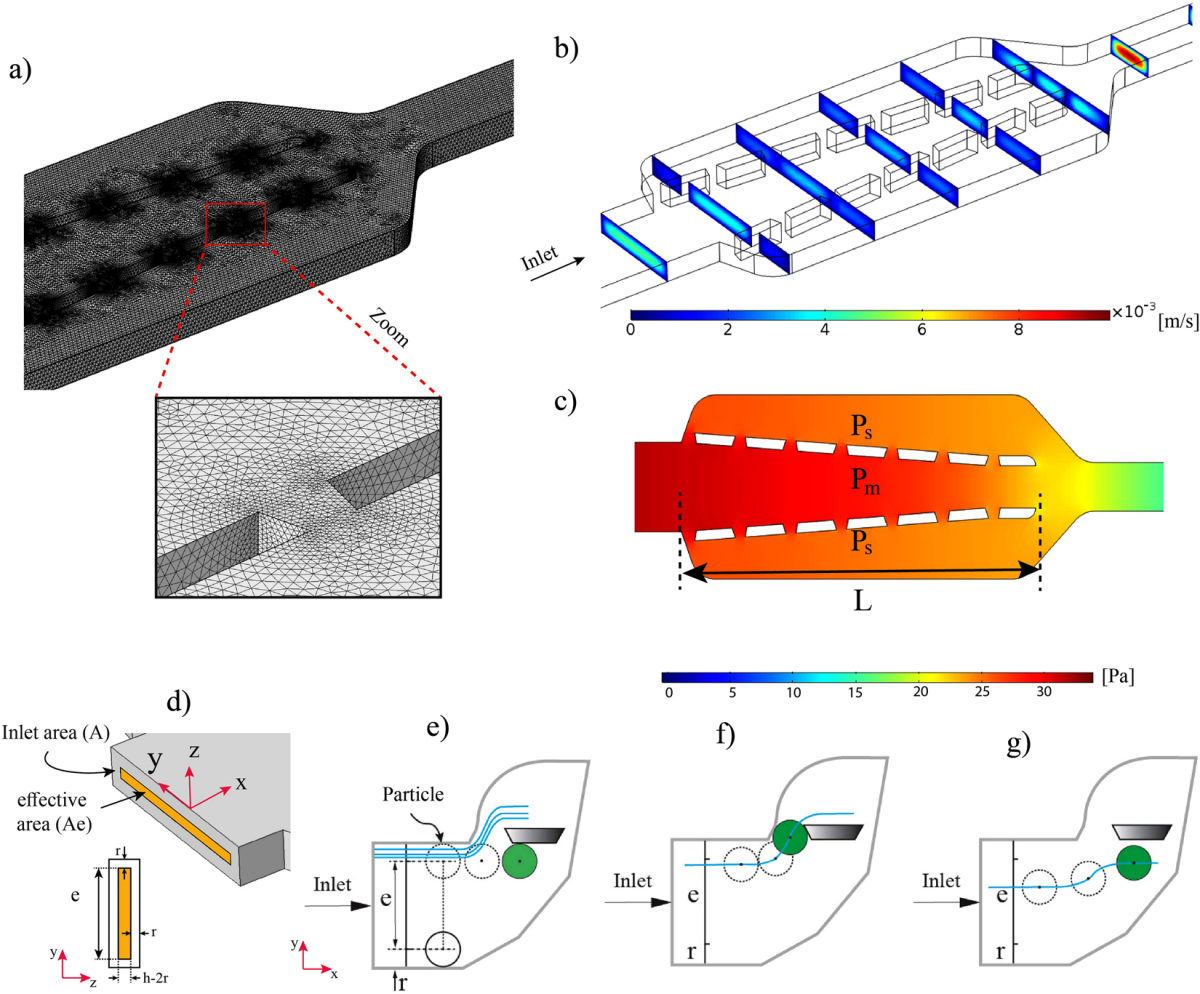
Laminar flow simulation within the microfluidic network with Design 3 under the inlet velocity of 2.8 mm/s, (**a**) mesh distribution in the model and traps: 1456469 tetrahedral elements were created to generate the mesh, (**b**) Velocity contour (m/s), (**c**) Pressure contour (Pa), (**d**) A schematic of the effective area ${A}_{e}=(h-2r)\times (D-2r)$ and ineffective area (A-Ae), (**e**) Flow streamlines within different regions of the inlet and their potential to navigate cells/particles into the trap sites. This diagram shows that streamlines flowing through the first trap cannot capture the very close particle to the sidewall. (**f**) A close streamline to the sidewall passing through the center of mass and can direct the particle into the first trap site. (**g**) Although the streamline passes through the center of mass, it is not able to direct the particle into first trap site. (The blue lines show the flow streamlines inside the main microchannel, h: height of the channel, e: effective entrance width, r: radius of the target particle or cell).

The set parameters were used to model the microfluidic in COMSOL Multiphysics and characterize the velocity contours and the pressure distribution within the microfluidic device. The pressure difference values between the main and side channels were computed for three different sidewall angles and plotted in Fig. 3a. This figure illustrates the pressure differences between the main channel *P*_*m*_ and the side channels *P*_*s*_ for three different angles under a constant inlet velocity (2.8 mm/s). It is evident that when inlet and outlet cross-sections are similar, for a straight channel and Design 1 (*θ* = 0), the pressure difference between the main channel and side channels is small and may be even a negative value (for trap 6 and 7), meaning that the flow may be reversed from the side channels into the main channel. Figure 3d,e present the flow streamlines and velocity field (normalized arrows) respectively within 6^th^ and 7^th^ traps, confirming that the flow is directed from side channels to the main channel. As two arrows show in Fig. 3d, the flow is trying to enter traps, but the gradient pressure is not strong enough to navigate flow completely to traps, and they re-enter to the main channel. By increasing the sidewall angle (*θ* = 4.4°), the pressure differences between the main and side channels are increased to remain positive for all trap sites, providing a flow direction toward the traps. For example, the pressure difference increases from 1.5 Pa to 2 Pa for the first trap (Design 3, *θ* = 4.4°). As can be seen in Fig. 3a, using the third design, the maximum positive pressure gradient between the main and the side channels can be reached. It can be observed that the effect of the stepped sidewall is noticeable in the middle of the channel while its effect is less pronounced on the first and last trap sites. Mesh distribution as shown in Fig. 4a was generated in order to simulate the fluid flow in the microfluidic channels. Pressure and velocity contours for Design 3 are shown in Fig. 4b,c, clearly indicating the pressure difference along the main channel. However, its values remain high compared to the side channels at the same positions. The pressure distribution shows that for all traps, there is a positive pressure gradient from the main channel to the side channel, directing the suspended particles to the trapping sites.

In order to characterize the effects of sidewall angle on the trapping efficiency of the system, flow passing through each trap site was calculated for three different designs using the geometric parameters shown in Table 2 and considering water with a dynamic viscosity of 0.001 Pa.s and density of 998 *kg*/*m*^3^ as the working fluid. Table 3 indicates the proportion of flow at each trap to the total flow entering the main channel from the inlet. The calculated values confirm a lower ratio at the seventh trap site, meaning that a low flow can pass through last traps, and as a result, the chance of cells to be captured by this trap is relatively low. Moreover, for the third design, these ratios are larger compared the two first designs. For Design 1 and 2, the passing flow rate for the last trap is negative (showing the reversed flow from side channel to main channel) while for Design 3 this value becomes positive (flow from the main channel to the side channels). It can be seen that more than 65% of the entering flow rate is drained by the outlet for Design 1 and its value is reduced to 36% for Design 3.

**Table 3:** Flow rate ratios for the three different designs (%).

Ratio	Q_1_/Q_in_	Q_2_/Q_in_	Q_3_/Q_in_	Q_4_/Q_in_	Q_5_/Q_in_	Q_6_/Q_in_	Q_7_/Q_in_	Q_out_/Q_in_
Design 1 (*θ* = 0)	9.29	5.07	2.53	1.18	0.40	−0.21(*)	−0.92(*)	65.32
Design 2 (*θ* = 2.4°)	9.63	6.83	4.15	2.63	1.59	0.55	−1(*)	51.24
Design 3 (*θ* = 4.4°)	10.41	8.06	5.40	3.87	2.74	1.45	0.13	36.00

*Q*_*i*_: Flow rate at *i*_*th*_ site, *Q*_*out*_: Flow rate at the outlet, *Q*_*in*_: Flow rate at the inlet.

*Flow is directed from the side channels to the main channel.

The concept of using a converging channel to enhance the trapping capability of the microfluidic traps is confirmed with the derived flow rate ratios. The narrower outlet opening increases the hydrodynamic resistance of the microchannel; however, the choice of a very narrow outlet is limited because of the clogging possibility within the channel. In order to have a continuous flow inside the main channel without clogging, the dimensions of the outlet should be carefully determined. Experiencing a less stress by the trapped particle/cell due to the hydrodynamic forces is another determining factor for designing the outlet width. By increasing the angle (*θ*), the fluidic resistance of the main channel is increased and more stress will be applied on the trapped particles/cells due to the higher pressure in the main channel. Due to these reasons, we limited the minimum outlet width of the main channel such that only three particles could pass through the outer channel easily at the same time to prevent the accumulation of particles and imposing very high stress on the trapped particles/cells.

As was mentioned earlier, the flow behavior inside the microchannel is laminar, so in order to trap particles into the designed sites, two conditions need be met. First, at least one of the streamlines has to pass through the center of mass of the entering particle which is supposed to be captured. Second, the streamline passing the center of the particle has to pass through one of the trap sites. Even though some streamlines very close to the sidewall of the inlet channel can pass through trap sites, they have no impact on particles since they cannot pass the particle center due to the physical limitation of the particle^19,30^. In fact, the streamlines at a distance less than the radius of particles from channel wall (both in z and y directions) are not able to deliver cells into trap sites since theses streamlines do not pass through the center of cells. This concept and the various possible conditions for streamlines and the injecting particles are clearly shown in Fig. 4(d–g). By considering the two above conditions and size of the target particles, more precisely, the trapping probability *T*_*e*_ for each trap (i) can be defined as the fraction of flow at the effective area which can pass through the traps. Effective area is a portion of the inlet area (A) where the passing streamlines are able to deliver particles into traps (*A*_*e*_ = (*D* − 2*r*) (*h* − 2*r*)).

$${T}_{e}=\frac{Flow\,rate\,passing\,through\,{i}_{th}trap\,coming\,from\,the\,effective\,area}{Total\,inlet\,flow\,at\,the\,effective\,area\,({A}_{e})}$$

Flow rate at each trap is a mix of two flow rates coming from the effective and the ineffective areas. In order to estimate the efficiency of a specific trap, the portion of flow rate from the effective area which enters into the trap needs to be determined. Because of the laminar flow, the flow rate corresponding to a certain section can be predicted by counting the number of streamlines passing through the section of interest. Since the particles entering in a laminar flow follow the streamline paths, the probability of a trap site can be approximated by measuring the ratio of the passed particles at the trap site to the total number of arriving particles at the effective entrance of the main channel.

**Figure 5:**
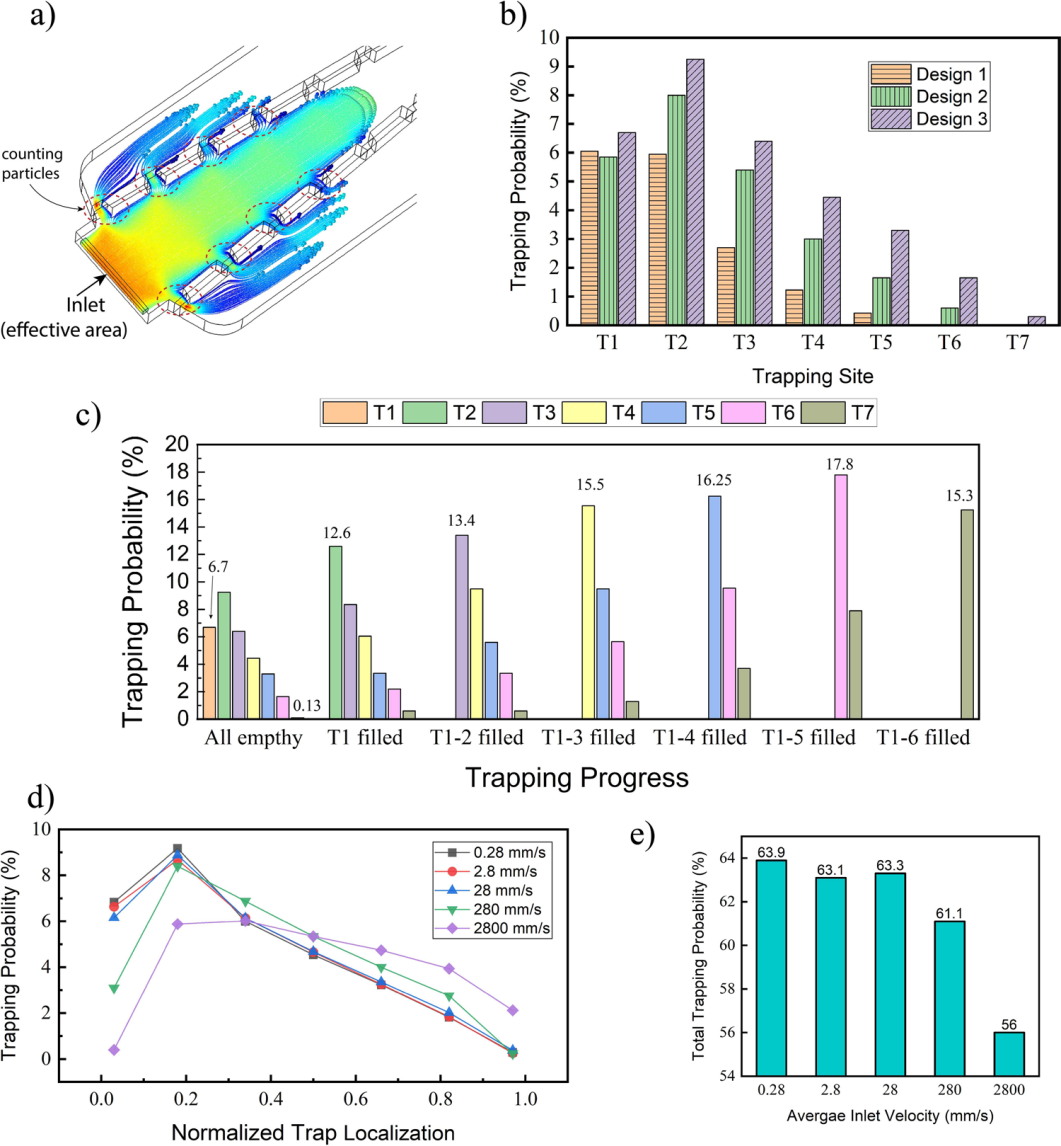
(**a**) Particles are released at the effective area, and the number of particles passing each trap is counted to estimate the trapping efficiency (contour color shows the velocity magnitude of particles), (**b**) Trapping probability of each trap site for three different designs (inlet velocity: 2.8 mm/s), (**c**) Trapping probability of each site during the trapping process (inlet velocity: 2.8 mm/s), when a trap site (*T*_*i*_) is filled, its trapping probability is considered to be zero, (**d**) Changing the trapping efficiency versus the normalized trap location (x/L) by increasing the inlet velocity (velocity in mm/s, x: trap location shown in Fig. 3, L = length of the converging channel), (**d**) Total trapping probability with respect to the inlet velocity. ($total\,trapping=2\sum _{0}^{7}{T}_{ei}$).

For this purpose, with the aid of the particle tracing module of COMSOL Multiphysics, a uniformly distributed number of particles representing the streamlines were released across the effective region (*A*_*e*_) of the main channel inlet and their trajectories were then simulated. It was assumed that particles do not affect the fluid flow in the channel. By monitoring the trajectory of each particle or cell, the efficiency of each site for trapping was evaluated by counting the particles flowing through it and comparing to the total number of the released particles. Figure 5b shows the probability of trapping for each trap using the mentioned concept. Due to the geometric symmetry of the design, the chance of trapping is identical for both trapping series on the right and left sides of the main channel ($T{e}_{1 \sim 7}\,(right)=T{e}_{1 \sim 7}\,(left)$) and due to this reason, the probability was calculated only for one trapping series. From this figure, it is clear that the trapping probability for each trap is almost proportional to its relative flow (Table 3). However, the results show that by considering the size of particles (effective area), the trapping efficiency for the second trap is higher compared to the first site. The differences between two values arise from the fact that for calculating the relative flow rate, the total inlet flow was considered while trapping probabilities were measured based on the total flow rate passing only through the effective area. Probability and relative flow rate for each trap have the same trend, and by increasing the hydrodynamic resistance of the main channel both the probability and the relative flow rate are increased.

**Figure 6:**
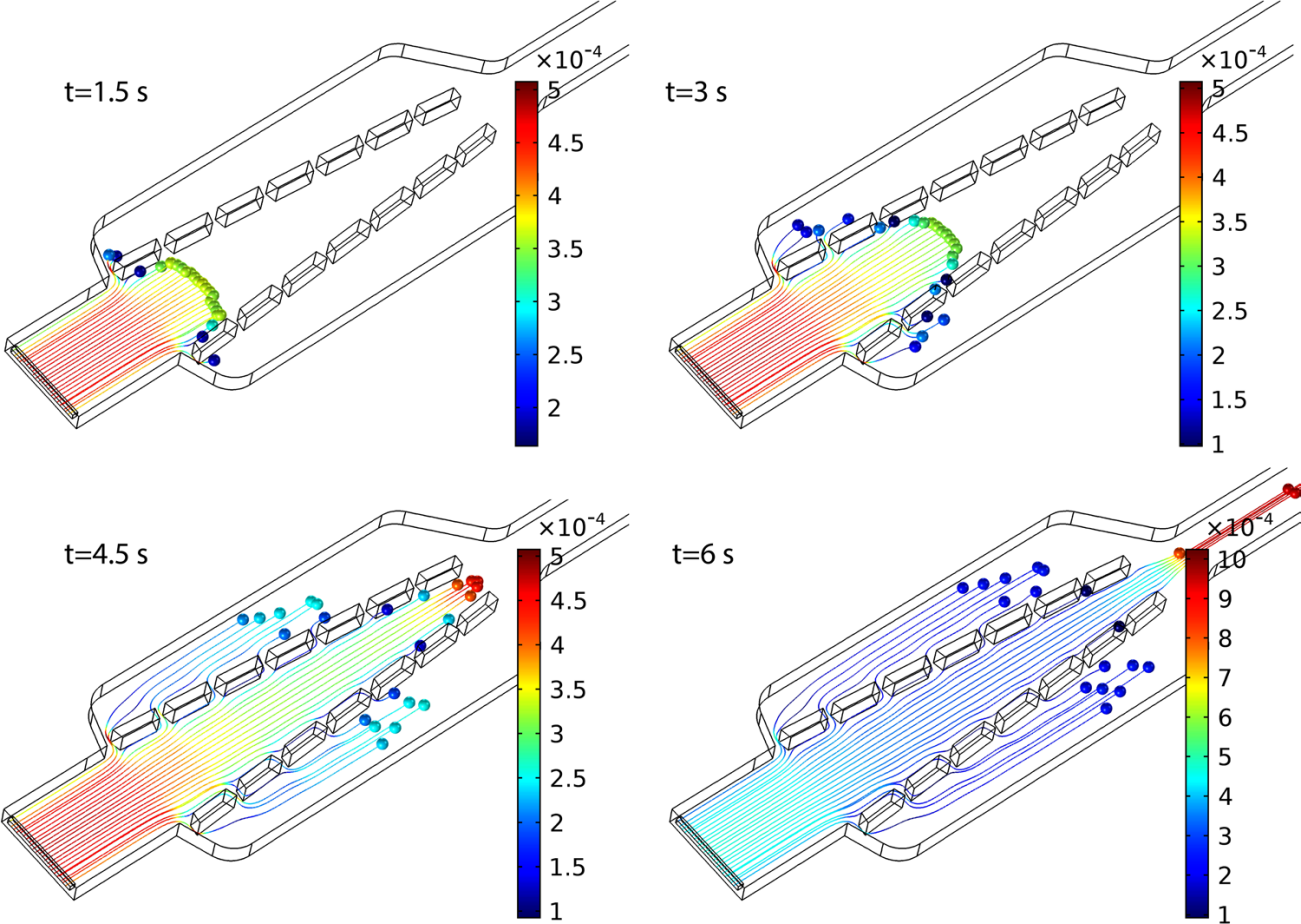
Trajectories of particles entering the main channel of the microfluidic device at different times. The particle tracing simulation was performed to predict the probability of trapping of each site. It should be noted that the particles in the simulation are virtual, so they are not stopped at the entrance of the trap sites. (Inlet velocity: 0.28 mm/s, the contour color shows the velocity magnitude in m/s).

For estimating the trapping efficiency, it was assumed that there are no particles in the channel. Once a trap is filled, the trapping efficiencies of the rest traps are changed due to the different fluidic resistance in the main channel and traps site. The effects of trapping progress on the probability of each site are shown in Fig. 5c. For measuring the probability values, it was assumed that trapping is occurring sequentially. When a trap is filled by a particle, its fluidic resistance is increased, allowing other traps to receive the subsequent particles with a higher probability. Based on the results, the average trapping probability for all traps is 13.93% with a standard deviation of 3.6, showing a fairly uniform trapping process. In order to determine the effects of different inlet velocity on the trapping performance of the system, the trapping probability for each trap site of the Design 3 was calculated by carrying out a similar simulation. When the inlet velocity was increased from 0.28 to 2.8 mm/s, the difference between trapping efficiencies was negligible. By increasing one order more of the inlet velocity, the trapping efficiency appeared to be decreased in the first position, but the efficiency did not change significantly at the other positions. By increasing the inlet velocity to 280 and 2800 mm/s, the trapping probability at the first and second positions decreased significantly while the trapping probability values at the 5^th^ and 6^th^ were increased as shown in Fig. 5d. However, the total trapping efficiency of the system was reduced to 61.1% and 56% showing 2% and 8% drops respectively compared to the other inlet flow values (Fig. 5e). According to eq. 1, by increasing the inlet velocity, the hydrodynamic resistances of both the main channel and the trap sites are increased, and the results show that at a higher inlet velocity, the equivalent hydrodynamic resistance of the trap sites is increased faster in comparison to the hydrodynamic resistance of the main channel, therefore, reducing the total trapping efficiency. It can be seen that the device can operate at a wide range of inlet velocity without fluctuations. Figure 6 demonstrates the time-dependent particle tracing simulation. A certain amount of the particles was released uniformly at the entrance of the main channel (only effective area) and their trajectories were observed through finite element simulation. As can be seen in this figure the particles have different velocities depending on their initial position at the inlet of the rectangular channel. At the middle of the channel, particles possess larger velocities and their velocities slow down as they arrive closer to the channel walls. Depending on the position of the particles, they can be, either trapped by one of the trap sites or drained by the outlet of the main channel.

### Mechanical stress analysis

The fluid-induced stress can alter the functional behavior of both the healthy and cancerous cells such as proliferation, transport, differentiation or even cell death^31–33^, therefore it is crucial to provide proper experimental conditions with reduced hydrodynamic stresses to avoid their undesired effects (mechanical damages and decreased cellular viabilities) on cells. When the cells or particles are far from the trap sites, they are subjected to a relatively low stress. After trapping, the particles/cells have to undergo much more mechanical forces due to the larger fluid pressure on their surfaces and their contacts with the traps. An additional FEA analysis was carried out considering a polystyrene single particle (microsphere with diameter 50 μm) sitting in the trapping site in order to characterize the mechanical stresses experiencing at the trap position. A fluid-structure interaction (FSI) analysis (details described in^32^) was created using the COMSOL Multiphysics to estimate the hydrodynamic stress acting at the fluid-particle interface and characterize deformation of the trapped single particles/cells due to the pressure difference across the cell. By assuming an incompressible and Newtonian fluid, the stress tensor acting at the interface of particle and fluid can be determined by the following formula^34^:

7 $${\sigma }_{ij}=-p{\delta }_{ij}+\mu (\frac{\partial {v}_{i}}{\partial {x}_{j}}+\frac{\partial {v}_{j}}{\partial {x}_{i}})$$

Where *σ*_*ij*_ is stress, *δ*_*ij*_ is the Kronecker delta, *v*_*i*_ is the fluid velocity in “*i*” direction, and *p* is the fluid pressure. Equivalent elastic strain (EQS) can also be used to quantify the deformation of cells/particles under hydrodynamic force^32^. In order to measure the mechanical stress and the hydrodynamic deformation, it was assumed that all traps are closed by particles, and the pressure and deformation on a cell trapped in the second site (having the maximum trapping probability based on Fig. 5b) was calculated by considering the physical and mechanical properties of polystyrene particles: density (1050 kg/m^2^), Elasticity (3 MPa) and Poisson ratio (0.33) and water as the working fluid. For FSI simulation, the contact points are also assumed to be fixed and no displacement happens in contact points of particles and trap walls, and on the boundary of particle and fluid, the velocity of fluid equals the rate of change for displacement of the solid domain (particle). Similar to the computational section, for FSI simulation, the inlet velocity and outlet pressure were set 2.8 mm/s and zero respectively and no-slip conditions were applied for all walls.

**Figure 7:**
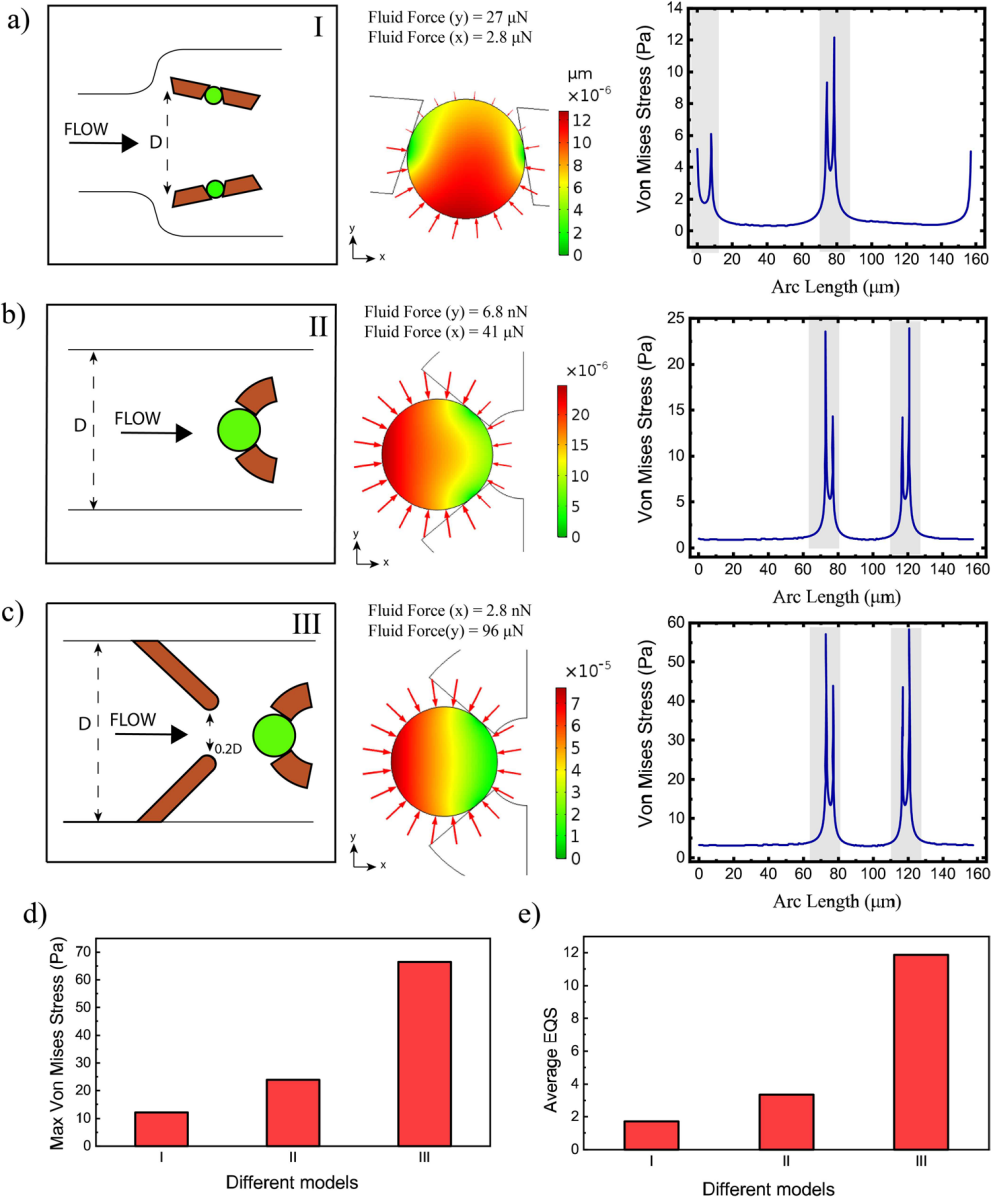
Total displacement (μm) contour due to the hydrodynamic forces, total stress on the trapped particle walls (red arrows), total fluid forces in x and y directions (by integrating total fluid stress on the whole perimeter of the trapped particles) and Von Mises stress along the perimeter of the trapped particle and the contact parts between particles and trap walls are highlighted: (**a**) in our Design (max. stress: 12.15 *N*/*m*^2^), (**b**) in the design in^35^ (max. stress: 23.91 *N*/*m*^2^), (**c**) in the design presented in^36^ (max. stress: 62.41 *N*/*m*^2^), (**d**) Maximum Von Mises stress (at the contact point between particles and trap walls), (**e**) average equivalent strain (EQS) over the trapped particles for different models. (Inlet velocity: 2.8 mm/s, the contour color on the micro particles shows the magnitude of the total displacement, and the red arrows show the distribution of hydrodynamic stress around the particles and their scale factors are not identical, scale factors are 20, 5 and 1.5 for models I, II and III respectively).

The FSI results (Fig. 7a) show that with the aid of this design, low stress and deformation are applied to the trapped particle/cell compared to the similar designs, suggesting that cells can be gently trapped by this micro device without experiencing a high stress. Considering the same boundary conditions, the same analysis was carried out to compare the applied stress and deformation by two other microsystems^35,36^, and the results are shown in Fig. 7b,c. The Von Mises stress was computed from stress tensor of the particle to estimate the reaction stress to fluid forces on the trapped particle including contact points between trap walls and particles (Fig. 7 right panel). It is evident that our design offered relatively a gentle trapping in comparison to the other two devices, under the same conditions, while is able to manipulate cells with a high efficiency. In our design, the maximum reaction stress is 12.15 Pa at the particle-trap wall points and is smaller in comparison to two other models. This figure also shows the total stress distribution around the trapped particles (red arrows) for our design and two previously reported works, showing that smaller total stresses are distributed around the trapped particles in our design compared two others. The maximum stress and average equivalent elastic strain (EQS)^32,34^ were also calculated and plotted in Fig. 7d,e respectively. The average equivalent elastic strains for each device was computed by averaging EQS over the total surface of the trapped particles. EQSs show that, under the same conditions, the deformation-induced by hydrodynamic forces is less in our design in comparison to the two other devices.

**Figure 8:**
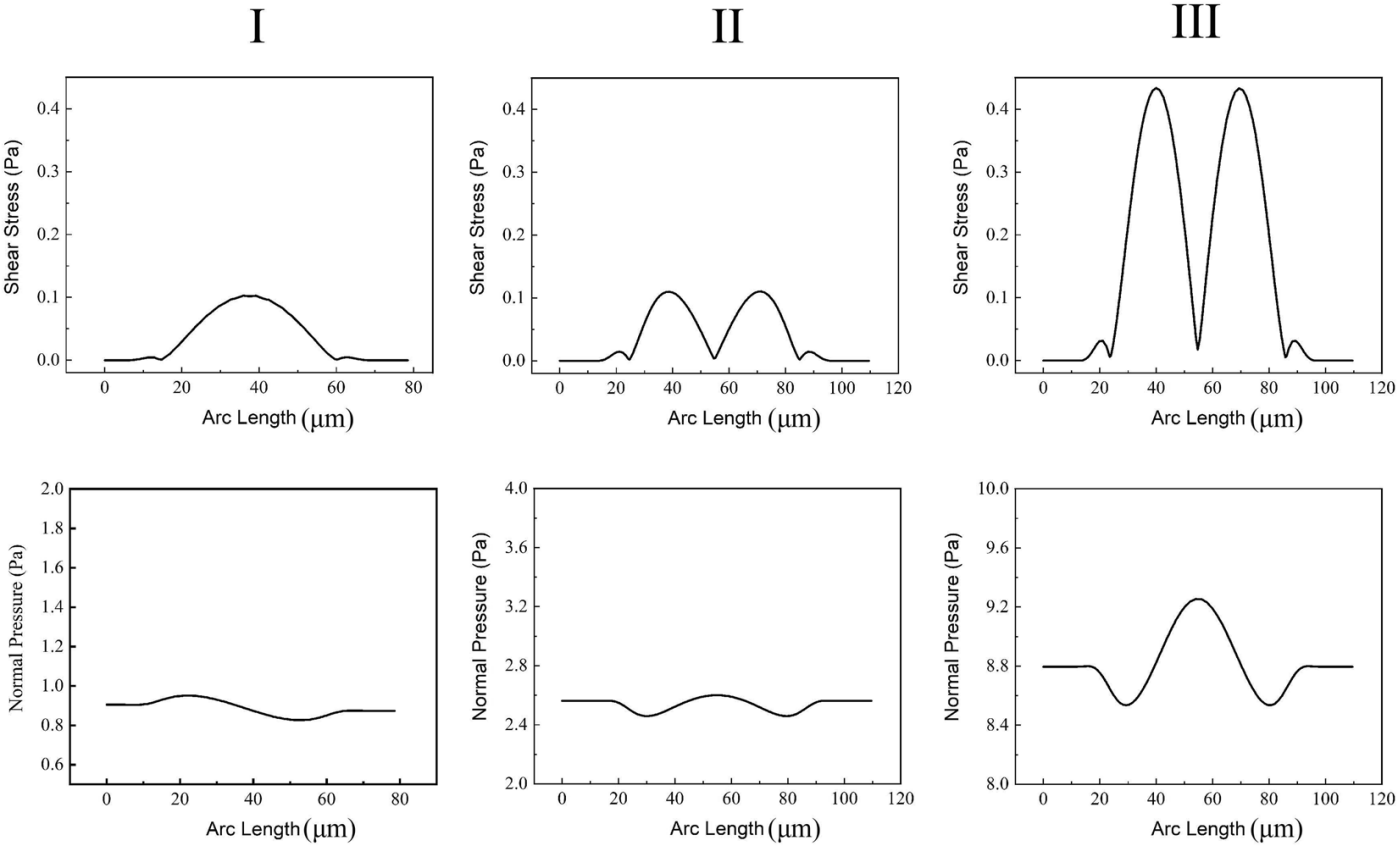
Shear stress and normal pressure applying on the trapped particles in the current design (I) compared to other designs (II and III) under the same boundary conditions (inlet average velocity: 2.8 mm/s and the outlet pressure set zero).

Two types of fluid force are acting on the particle wall at the boundary of the fluid domain: normal forces due to hydrodynamic pressure and tangential forces due to shearing effects. Under the same boundary conditions, the shear stress and normal pressure applying on the wall of trapped particles for our model and two other models are plotted in Fig. 8. As can be seen in this figure, the normal pressure on the particles wall is much more for all three models compared to the experienced shear stress due to the fluid flow. In our design less normal pressure (P_max_: 0.95 Pa) is applied to the trapped particle while in model II and III, the trapped particles experience 2.6 Pa and 9.2 Pa normal pressure respectively. In the current model, the maximum shear stress on the trapped particle is slightly smaller than the second model while is much smaller than the third model. The total force applied by only fluid flow on the trapped particles is reported in Fig. 7 in both x and y directions, and compared to other models, in our design less total stress is applied to the particle wall.

So far, the design parameters of the microfluidic device were optimized, and the trapping performance of the microsystem was analyzed using the simulation. The optimal parameters were used for following experiments and further analysis.

### Experimental results

**Figure 9:**
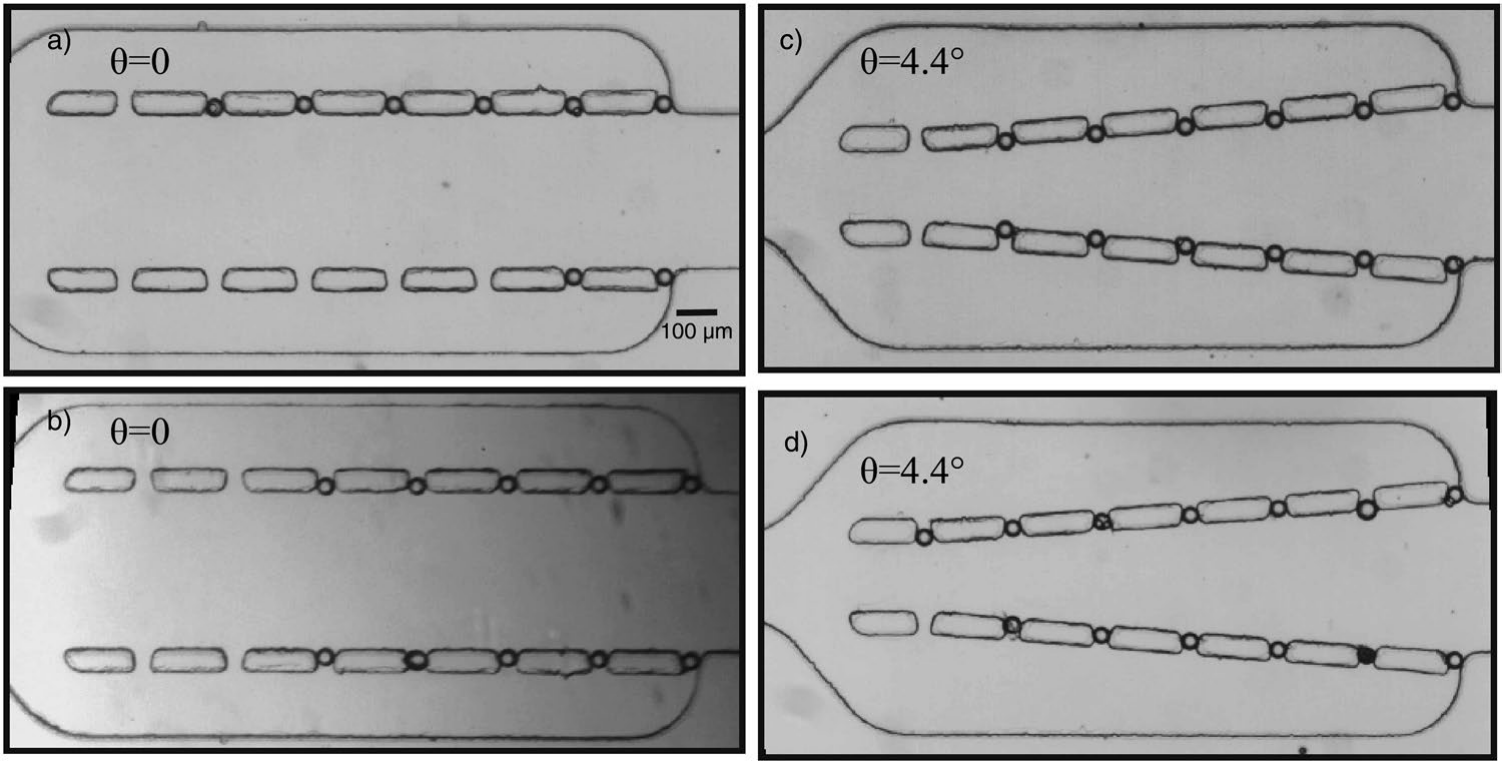
Microscopic images of single particle trapping: the performance of the microsystem to capture biological particles for Design 1 (**a**,**b**) and Design 3 (**c**,**d**) under the same conditions. (Fluid is flowing from right to left).

After optimizing the geometrical parameters of the microfluidic circuit, the optimized microfluidic device with different sidewall angles was used to test the trapping performance of the microsystem using microbeads with a diameter of 50 μm. Under the flow with a very low Reynolds number (Re ≤ 1), it is expected that particles move on the streamlines in agreement with the simulation results. Although trapping efficiency is not influenced by the inlet flow rate for a very low Reynolds number (based on the numerical simulation), the microparticles were loaded with a relatively low flow rate into the channel to observe the motion of the particles (<10 μl/s). In order to avoid clogging inside the microfluidic channel and monitor trajectories of particles, they were injected at a low concentration, and it was found that microbeads are distributed non-uniformly at the entrance of the main channel and non-sequential trapping was observed during micro particles loading. In the simulation section, the fully developed fluid flow at the entrance section was used to estimate the efficiency of the system. The entrance length (the distance before the fluid flow becomes fully developed) can be obtained from L_e_ = 0.006Re.D_h_, where Re is Reynolds number and D_h_ is the hydraulic diameter of the channel^32,37^. By considering the hydraulic diameter of the entrance channel and calculating the Reynolds number, the entrance length is very small. The distance of the channel before the inlet section is much longer than L_e_, confirming that in our experiment, at the entrance section always the fluid flow is fully developed. Three different designs were tested to observe their ability to trap particles and characterize their efficiency. It was observed that the main channel convergence affects significantly the efficiency of single particle trapping. Figure 9 shows the optical images of the trapped particles for both Design 1 and Design 3 under the same conditions after 15 min of operation, confirming the feasibility of the microdevice for trapping single cell/particle. As can be seen from the microscopic images, more particles were captured by the trap sites using Design 3 compared to Design 1. By repeating the injecting process of particles using new and empty microfluidic devices, it was observed that more than 90% of Design 3’s traps achieve single-particle trapping. Furthermore, by reversing the infusion of the medium, the device was shown to be capable to release the most of the trapped particles.

**Figure 10:**
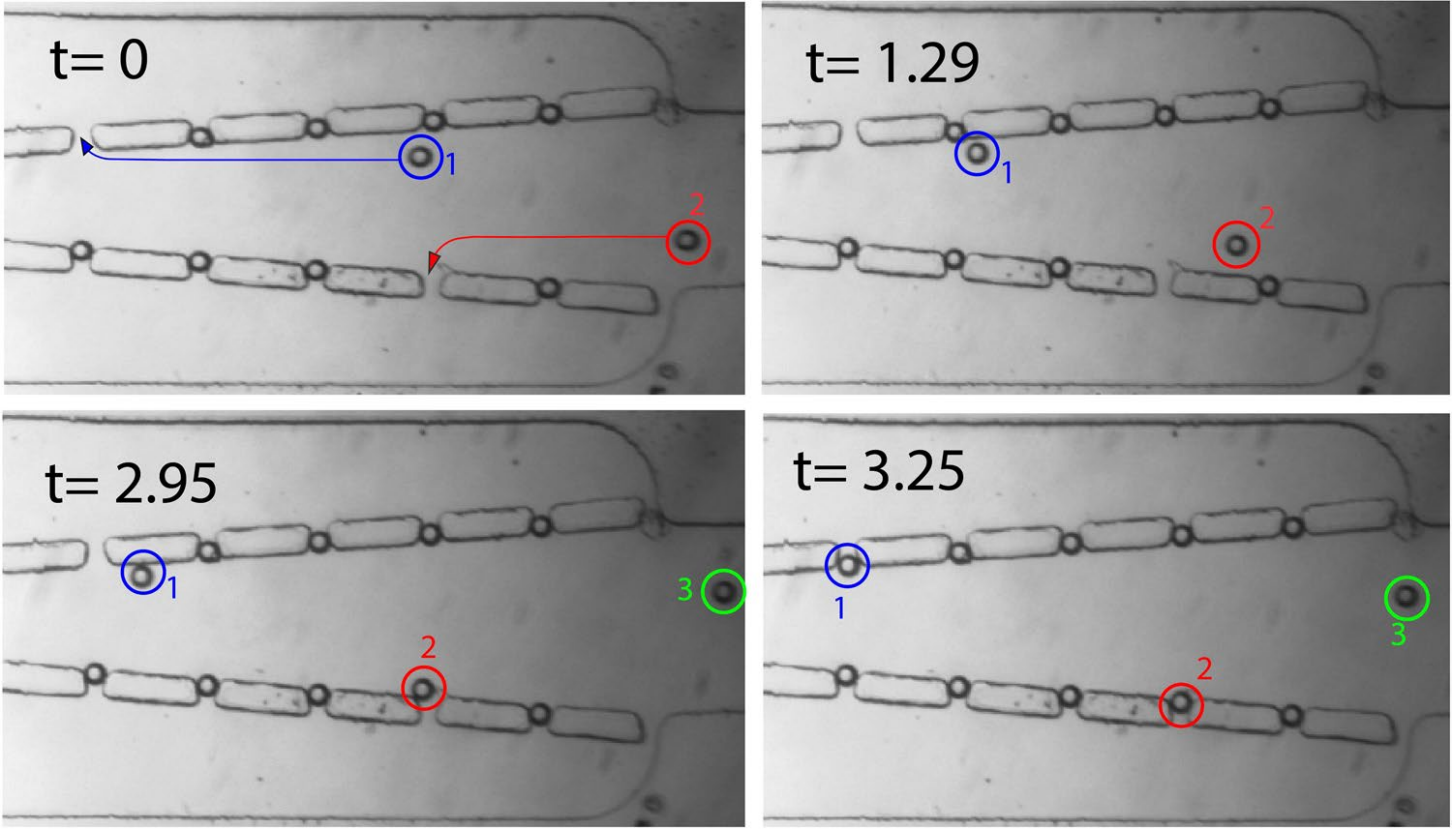
Time-dependent motion of particle within the microchannel under a very low flow rate to observe their motions. Particle 1 due to its initial position is flown toward a trap on its right side while particle 2 is flown toward a trap site on its left side because of its initial position. (Fluid is flowing from right to left).

Figure 10 illustrates the time-dependent position of two particles (1 and 2) under a fairly low inlet velocity. As mentioned earlier, the particles may navigate to one of the trap sites depending on their starting positions in the entrance cross-section of the main channel. As it can be seen from the figure, some of the trap sites have already been filled with particles, and the extra hydrodynamic resistance induced by the immobilized particles diverge the flow streams and the motion of the subsequent micro-particles entering to the system toward the other vacant trapping sites. Particle 1 moves toward the empty trap site on the top while particle 2 is directed toward the empty site on the bottom side channel and finally they are trapped. The simulation results showed that by decreasing the outlet width, more flow could pass through all trap sites, but microbead clogging occurs during the injecting process, inducing significantly more stress on the immobilized particles. It was observed that in the case of Design 3, microbead clogging does not occur as extra micro particles can be drained easily by the outlet. Due to the low flow rate at sites close to the outlet, their chances to direct particles toward trap sites is very low.

**Figure 11:**
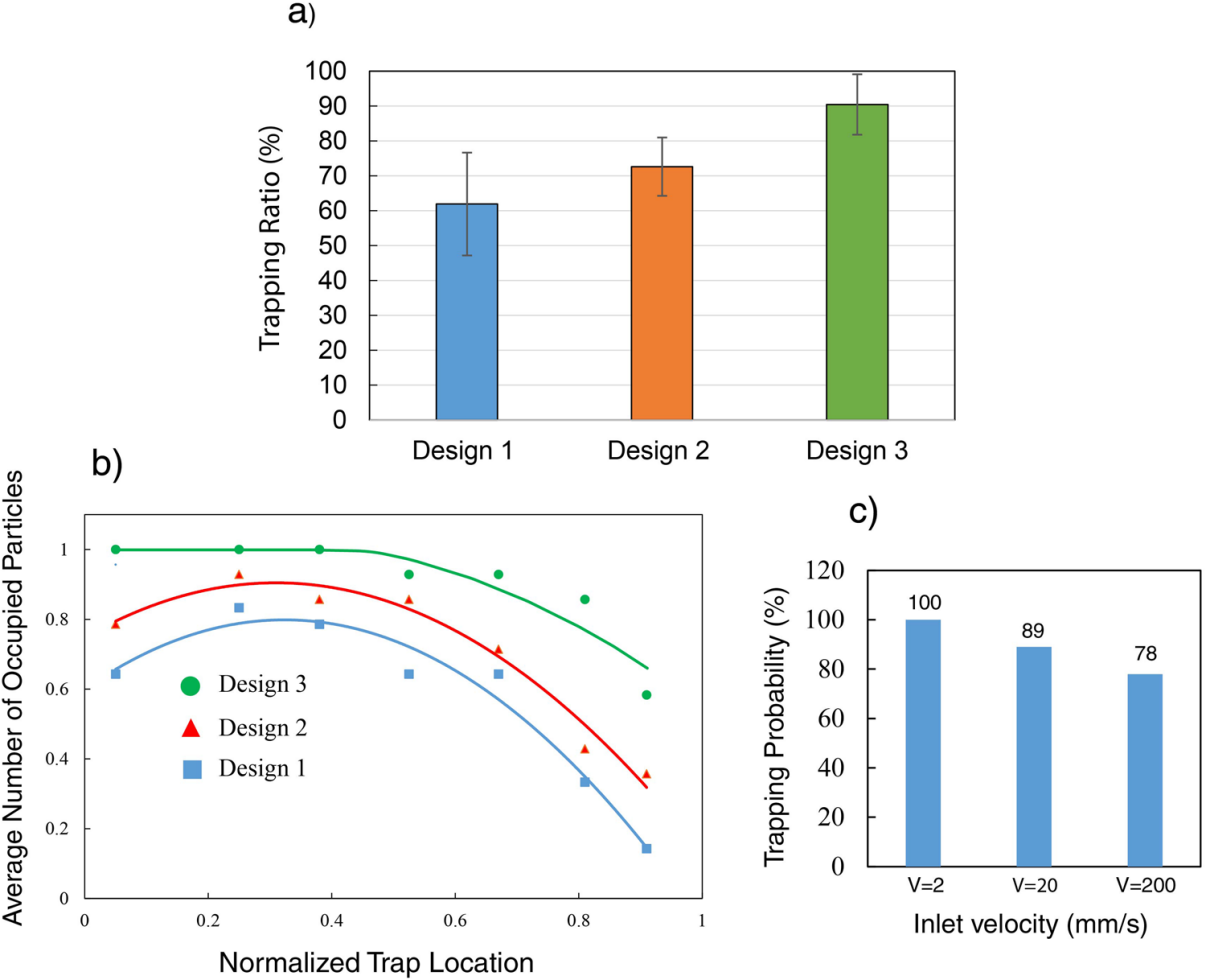
(**a**) Trapping probability of each design under the same conditions, (**b**) Trapping efficiency versus normalized trap location (x/L) for different designs (solid lines show the curved trends, L: length of the converging channel, x: trap location), (**c**) trapping efficiency for the first trap (Design 3) by increasing the inlet velocity.

To assess and quantify the trapping efficiency of each microfluidic system, the ratio of the number of trapped particles to the total number of sites was calculated. For each device, the experiment was done at least six times, and the average value was calculated and reported as the trapping efficiency. In order to ascertain that similar conditions exist for all experiments, a new device, free of particles, was used. To characterize the trapping efficiency of each device, spherical 50 μm polystyrene micro particles were injected through the syringe pump at the velocity of 2 mm/s then the highest number of occupied traps was recorded. Figure 11a shows the trapping efficiency of three different microfluidic systems. The results show that the total trapping efficiency of the system with identical inlet and outlet openings is about 60% and this value can be increased up to 90% by tuning the convergence of the main channel. In agreement with the numerical analysis, it was observed that trap sites close to the outlet remain empty even after 15 min of operation due to the low rate of flow. However, in Design 3, the gradient pressure at the sites is improved and particles will be captured with a higher probability. Moreover, to quantify the trapping ability of each trap, the average number of particles trapped in different traps were recorded (this value is 1 for a site location once the trap is filled by particles, and zero when is remained empty during the experiment). The experimental trapping efficiency of each trap location is presented in Fig. 11b. For Design 1 and 2, in agreement with the finite element analysis, it was observed that the average trapping efficiencies for traps *T*_6_ and *T*_7_ are almost low, confirming their low flow rate ratio values, and the efficiency is decreasing from *T*_1_ to *T*_7_. However, for Design 3 the average trapping is higher compared to the other designs and more uniform. The average trapping for first, second and third trap locations are almost similar for Design 1 and Design 2, and it reaches to maximum for Design 3. For the fifths, sixth and seventh trap locations, the average trapping is significantly improved from Design 1 to Design 3. For 7th trap location, the average trapping is increased by almost 50% which is in good agreement with numerical simulation indicated in Fig. 6b. In order to observe the effects of the inlet velocity on the trapping, the trapping efficiency for the first trap (Design3) was experimentally measured by increasing the inlet velocity to 20 mm/s and 200 mm/s. We observed that by increasing the velocity, the efficiency of the first trap is slightly reduced by 11% and by increasing the velocity to a higher velocity (200 mm/s), the efficiency of the first trap reached 78% (Fig. 11c) and its trend is in agreement with the simulation results. It was observed that by increasing the inlet velocity, the traps located at the middle part of the main channel (T2–4) are filled at an earlier time compared to the first trap.

## Conclusions

In this study, a microfluidic platform was developed to capture single particles using a hydrodynamic-based approach and the fluidic resistance concept. A series of trap sites were located on both sides of the main channel to accommodate single particles by controlling hydrodynamic forces. In our design in order to increase the hydrodynamic resistance of the main channel and direct suspended particles or cells into the trap sites, inclined sidewalls were designed instead of extending the main channel length, resulting in a more compact configuration. Numerical simulations were performed to tune geometric parameters and optimize the trapping ability of the microsystem through estimating the trapping efficiency of the device. With the aid of this microsystem, single particles can be trapped rapidly, with the efficiency of 90%. Moreover, the numerical simulation revealed that single particles can be trapped at the trapping positions without experiencing a high level of stress in comparison to similar devices, suggesting a proper configuration for capturing delicate single cells. This system can be further developed for particle sorting by accurately designing and controlling the width of three outlets to allow all the small particles to be directed into two side outlets after passing through trap sites.
